# Isoniazid prophylaxis differently modulates T-cell responses to RD1-epitopes in contacts recently exposed to *Mycobacterium tuberculosis*: a pilot study

**DOI:** 10.1186/1465-9921-8-5

**Published:** 2007-01-27

**Authors:** Delia Goletti, M Pasquale Parracino, Ornella Butera, Federica Bizzoni, Rita Casetti, Duilio Dainotto, Gianfranco Anzidei, Carla Nisii, Giuseppe Ippolito, Fabrizio Poccia, Enrico Girardi

**Affiliations:** 1 Translational Research Unit, Department of Experimental Research, Istituto Nazionale Malattie Infettive Lazzaro Spallanzani, IRCCS Rome, Italy; 2 Clinical Epidemiology Unit, Department of Experimental Research, Istituto Nazionale Malattie Infettive Lazzaro Spallanzani, IRCCS Rome, Italy; 3 Cellular Immunology Unit, Department of Experimental Research, Istituto Nazionale Malattie Infettive Lazzaro Spallanzani, IRCCS Rome, Italy; 4 Presidio Interzonale di Pneumologia ASL Roma E, Rome, Italy; 5 Pediatric Unit, Health Department, Istituto Nazionale Malattie Infettive Lazzaro Spallanzani, IRCCS Rome, Italy; 6 Epidemiology Unit, Department of Experimental Research, Istituto Nazionale Malattie Infettive Lazzaro Spallanzani, IRCCS Rome, Italy

## Abstract

**Rationale:**

Existing data on the effect of treatment of latent tuberculosis infection (LTBI) on T-cell responses to *Mycobacterium tuberculosis *(MTB)-specific antigens are contradictory. Differences in technical aspects of the assays used to detect this response and populations studied might explain some of these discrepancies. In an attempt to find surrogate markers of the effect of LTBI treatment, it would be important to determine whether, among contacts of patients with contagious tuberculosis, therapy for LTBI could cause changes in MTB-specific immune responses to a variety of RD1-antigens.

**Methods and results:**

In a longitudinal study, 44 tuberculin skin test^+ ^recent contacts were followed over a 6-month period and divided according to previous exposure to MTB and LTBI treatment. The following tests which evaluate IFN-gamma responses to RD1 antigens were performed: QuantiFERON TB Gold, RD1 intact protein- and selected peptide-based assays. Among the 24 contacts without previous exposure that completed therapy, we showed a significant decrease of IFN-gamma response in all tests employed. The response to RD1 selected peptides was found to be more markedly decreased compared to that to other RD1 antigens. Conversely, no significant changes in the response to RD1 reagents were found in 9 treated subjects with a known previous exposure to MTB and in 11 untreated controls.

**Conclusion:**

These data suggest that the effect of INH prophylaxis on RD1-specific T-cell responses may be different based on the population of subjects enrolled (recent infection versus re-infection) and, to a minor extent, on the reagents used.

## Background

According to the World health Organization, one-third of the world's population harbours *Mycobacterium tuberculosis *(MTB) in an asymptomatic, latent form (latent tuberculosis [TB] infection [LTBI]) but retains a lifelong risk of future disease. The control and elimination process of the global TB epidemic could be enhanced by identification and treatment of individuals with LTBI, in particular of individuals who recently acquired the infection as the risk of developing active disease is higher in the first 2 years after exposure [1,2].

Until recently, the tuberculin skin test (TST) has been the only tool used to detect LTBI, but this test is flawed operationally and in terms of specificity and sensitivity [3]. Lately, *in vitro *assays have been made available that detect interferon (IFN)-gamma responses to a combination of antigens (early secreted antigenic target 6 [ESAT-6] and culture filtrate protein 10 [CFP-10]) encoded by the RD1 (region of difference) genomic segment, which is absent from most non-pathogenic mycobacteria, including Bacillus Calmette Guerin (BCG) [3-5]. Available evidence suggests that these tests may be more accurate than TST in the diagnosis of MTB infection, either latent or active, and two tests have been approved for the diagnosis of TB [6-13]. Moreover, results of our previous studies suggest that IFN-gamma response to multiepitopic peptides from ESAT-6 and CFP-10 (RD1) proteins selected by computational analysis is associated with active TB disease [14-18].

Several unresolved issues remain on the potential clinical use of IFN-gamma release assays [6,7], and one area of controversy is whether these immune assays can be used for monitoring the response to TB treatment. Conflicting results are available on the effect of therapy for active TB disease on RD1 responses with reduced or increased responses during treatment [15,19-21]. The existing data on the effect of LTBI treatment on T-cell responses are also contradictory [22-25]. One study shows declining responses [22], whereas others have shown unchanging [23], fluctuating or increasing responses during therapy [24,25]. It is plausible that variations in technical aspects of assay performance such as antigens used (proteins vs. peptides), assay formats (ELISA vs. ELISPOT), time of observation (during therapy or after therapy completion), and population studied (recent versus old contacts) might explain some of these discrepancies.

Consequently, we reasoned that in an attempt to find surrogate markers of the effect of LTBI therapy, it would be important to determine in a prospective study whether, in a defined population of recent healthy close contacts of patients with pulmonary TB, isoniazid (INH) for LTBI treatment could cause changes in MTB-specific immune responses to a variety of RD1 antigens, such as the RD1 overlapping peptides of the QuantiFERON TB-Gold assay (QTF-G), RD1 intact proteins and selected peptides. Moreover to evaluate whether or not a previous infection could influence such response we included a control group composed of subjects with a known past exposure to MTB.

## Materials and methods

### Patient population and study design

In a one-year period we enrolled close contacts of infectious TB patients in 2 outpatient services in Rome, Italy (Presidio interzonale di pneumologia ASL Roma E, and National Institute for Infectious Diseases "L. Spallanzani"). We included in the present analysis individuals who tested TST^+^, who consented to provide a blood sample at the time of initial screening (time 1) and on subsequent occasions within the following six months (after 1–2 months: time 2; after 6 months: time 3), and who were symptom-free and with normal chest radiographs [chest radiographs were read by one board-certified radiologist and at least one infectious disease specialist or pneumologist (DG, GA, DD)] on initial screening. Patients were not included in the analysis if they were at risk for Human Immunodeficiency Virus infection (HIV), or if they reported having had active TB in the past, or having previously received treatment for LTBI. Upon enrolment demographic and epidemiological information were collected, including information about BCG vaccination. Data were collected by the physician through a structured questionnaire.

Individuals included in the analysis were classified into four groups based on the history of past exposure to a patient with contagious TB, and on whether or not they were receiving treatment for LTBI. The study was approved by the ethics committee at our institution and all enrolled individuals provided written informed consent prior to screening procedures, as did the parents of the children included in the study.

### TST

TST was administered by the Mantoux procedure using 5 IU of purified protein derivative (PPD) (Chiron, Siena, Italy). Results were read after 72 hours. Induration of at least 5 mm was considered a positive response [26,27].

### Whole blood ELISA (WBE) based on RD1 selected peptides and proteins

Selection of Human Leukocyte Aplotype (HLA)-class II-restricted epitopes of ESAT-6 and CFP-10 proteins was performed by quantitative implemented HLA peptide-binding motifs analysis [14]. Peptides were synthesized as free amino acid termini using Fmoc chemistry (ABI, Bergamo, Italy). The following lyophilized peptides, diluted in DMSO at stock concentrations of 10 mg/mL, were used and stored at -80°C: ESAT-6 _6–28, 67–79 _and CFP-10 _18–31, 41–68, 74–86_.

All samples were analyzed by WBE assays, as previously described [16]. Briefly, 0.5 ml per well of heparinized blood was seeded in a 48-well plate and treated with RD1 intact proteins at 0.2 μg/ml (Lionex, Braunschweig, Germany), RD1 selected peptides (pool of CFP-10 peptides at 6 μg/ml; pool of ESAT-6 peptides at 10 μg/ml), purified protein derivative (PPD) at 5 μg/ml (batch RT 47, Staten Serum Institut, Copenhagen, Denmark) and Phytohemagglutinin (PHA) at 5 μg/ml (Sigma, St Louis, MO, USA). Samples were then incubated for 24 hours at 37°C. IFN-gamma levels in culture supernatants were assessed by a commercially available kit (QuantiFERON-CMI kit, Cellestis Limited, Carnegie, Victoria, Australia). For IFN-gamma values above 10 IU/ml serial dilutions of plasma were performed.

Results are presented as IU/ml for ELISA after subtraction of the appropriate control according to the described criteria [16]. Cut-off values were determined by constructing a Receiver Operator Characteristic (ROC) curve by means of LABROC-1 software and were 0.7 IU/mL for all stimuli.

### Commercially available assay

QTF-G (Cellestis Limited, Carnegie, Victoria, Australia) was performed and its results were scored as indicated by the manufacturer (cut-off value for a positive test was 0.35 IU/ml). For IFN-gamma values above 10 IU/ml serial dilutions of plasma were performed.

### Statistical analysis

The main outcome of the study was the effect of treatment in terms of IFN-gamma production in response to antigenic stimulation in the QTF-G and WBE, expressed as dichotomous (positive/negative) and continuous (IU/mL) measures. IFN-gamma mean ± SE was calculated. The Mann-Whitney U test was used to compare continuous variables, and Chi square or McNemar tests were used for categorical variables. Analysis was carried out with SPSS v 14 for Windows (SPSS Italia srl, Bologna, Italy).

## Results

**Figure 1:**
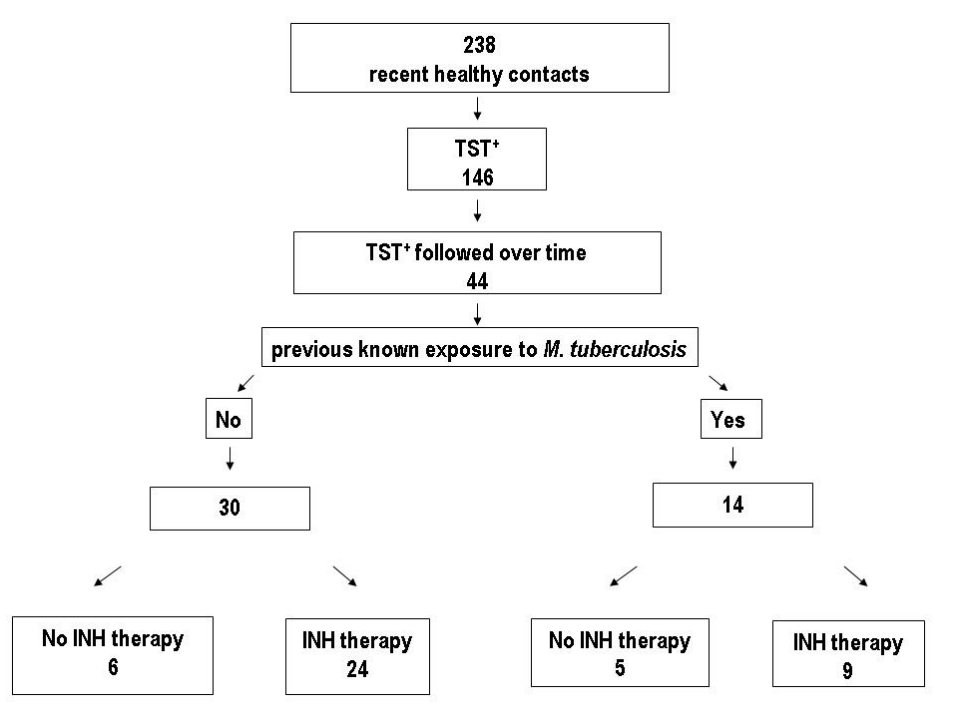
**Study flow diagram**. PPD: purified protein derivative; RD: region of difference; QTF-G: QuantiFERON TB Gold; INH: isoniazid; TST: tuberculin skin test.

We prospectively recruited 238 contacts of patients with sputum smear positive pulmonary TB. Among them, 146 resulted TST^+ ^and 44 of them accepted to be followed over time and were included in the analysis. The study group was divided into those who did not report having had any previous exposure to a smear positive TB case (30), and those who did (14). Among the former group, 6 patients did not receive therapy on the grounds that two were contacts of a patient with multidrug-resistant TB, two had chronic hepatitis C and two refused treatment; five subjects belonging to the latter group decided to not undergo INH therapy (figure 1).

Characteristics of individuals enrolled, divided according to previous TB exposure and LTBI treatment are shown in table 1. The group of those without a previous exposure under INH therapy had a significantly younger age (p < 0.001) compared to the other groups. No significant differences among the four groups were observed for the characteristics considered.

**Table 1:** Epidemiological and demographic characteristics of TST^+ ^contacts of pulmonary TB cases included in the study.

**Characteristics**	**No INH**		**INH**		
	**No past exposure** **N. 6**	**Past exposure** **N. 5**	**No past exposure** **N. 24**	**Past exposure** **N. 9**	**Total** **N.44**

**Median age (years)**	35.5	49	21	52	30
**Female gender N. (%)**	1 (17)	3 (60)	16 (66)	6 (66)	26 (59)
**Index cases, N.**	5	5	8	8	26
**Country of birth, N. (%)**					
Italy	4 (66)	5 (100)	13 (54)	8 (89)	30 (68)
Abroad	2 (34)	-	11 (46)	1 (11)	14 (32)
**BCG vaccinated, N. (%)**	2 (33)	1 (25)	11 (46)	1 (11)	18 (41)
**TST-response (induration), N.(%)**					
5–10 mm	1 (16)	-	7 (29)	1 (11)	9 (20)
11–15 mm	2 (33)	1 (20)	9 (37.)	1(11)	13 (30)
>15 mm	3 (50)	4 (80)	8 (33)	7 (78)	22 (50)

### Time course of MTB-specific immune response in recently exposed contacts

#### Untreated subjects

In all 6 untreated individuals without a known exposure to MTB IFN-gamma production did not significantly change over time for any of the stimuli and tests used (PPD, QTF-G, RD1 intact proteins and peptides) (table 2). Similar data were obtained in 5 individuals with a reported TB exposure in the past re-exposed recently to MTB that refused INH therapy (table 3).

**Table 2:** TST^+^ subjects without a past MTB exposure: trends of RD1 test during follow up in the responders*

**Group of subjects analysed**	**Number of subjects** **N (%)**	**Time 1** **0 months**	**Time 2** **1–2 months**	**Time 3** **6 months**	**Time 2 vs time 1**	**Time 3 vs time 1**
		**IFN-gamma (IU/ml)**				

**No INH**	6 (100)					
PHA	6 (100)	9.9 ± 4.3	8.6 ± 4.1	12.1 ± 6.5	Ns	Ns
PPD	6 (100)	27.4 ± 3.1	21.4 ± 4.9	23.5 ± 3.8	Ns	Ns
QTF-G	6 (100)	21.1 ± 5.2	19.5 ± 7.2	20.2 ± 6.1	Ns	Ns
RD1 proteins	6 (100)	16.1 ± 4.5	16.3 ± 7.2	7.3 ± 2.2	Ns	Ns
RD1 peptides	6 (100)	7.5 ± 3.3	7.0 ± 4.7	5.7 ± 4	Ns	Ns
						
**INH therapy**	24 (100)					
PHA	24 (100)	14.7 ± 2.7	16.8 ± 3	14.8 ± 2.8	Ns	Ns
PPD	24 (100)	17.6 ± 2.8	15.6 ± 2.7	14.1 ± 2.5	Ns	Ns
QTF-G	19 (79)	17.5 ± 2.7	11.6 ± 2.5	5.2 ± 1.3	p = 0.03	p = 0.0001
RD1 proteins	18 (75)	12.5 ± 2.6	5.4 ± 1.4	2.6 ± 0.9	p = 0.001	p = 0.0002
RD1 peptides	15 (63)	9.2 ± 1.7	3.4 ± 0.9	0.9 ± 0.1	p = 0.005	p = 0.0005

*Responders are defined as those responding to the tests at baseline.

T1: time 1 (baseline); T2. time 2 (after 1–2 months INH therapy), T3: time 3 (after 6 months, at therapy completion). IFN: interferon; PHA: Phytohemagglutinin; PPD: purified protein derivative; RD: region of difference; QTF-G: QuantiFERON TB Gold; IFN: interferon; IU: international units. INH: isoniazid; ns: not statistically significant.

**Table 3:** TST^+^ subjects with a past MTB exposure: trends of RD1 test during follow up in the responders*

**Group of subjects analysed**	**Number of subjects** **N (%)**	**Time 1** **0 months**	**Time 2** **1–2 months**	**Time 3** **6 months**	**Time 2 vs. time 1**	**Time 3 vs. time 1**
		**IFN-gamma (IU/ml)**				

**No INH**	5 (100)					
PHA	5 (100)	14.2 ± 3.4	17.1 ± 4.5	17.5 ± 4.6	Ns	Ns
PPD	5 (100)	31.4 ± 2.5	33 ± 3.4	29.3 ± 4.5	Ns	Ns
QTF-G	5 (100)	16.4 ± 5.1	12.3 ± 4.4	16.3 ± 6.7	Ns	Ns
RD1 proteins	5 (100)	11.8 ± 3.8	8.9 ± 3.2	13.5 ± 6.1	Ns	Ns
RD1 peptides	5 (100)	6.1 ± 2	2.2 ± 0.5	4.6 ± 2	Ns	Ns
						
**INH therapy**	9 (100)					
PHA	9 (100)	17.7 ± 6	20.4 ± 4.4	17.8 ± 4.1	Ns	Ns
PPD	9 (100)	19.6 ± 4.5	19.4 ± 4.7	24.2 ± 5.2	Ns	Ns
QTF-G	9 (100)	15.2 ± 4	11.3 ± 4	9.4 ± 2.7	Ns	Ns
RD1 proteins	9 (100)	6 ± 2	8.8 ± 3.3	11.9 ± 5.2	Ns	Ns
RD1 peptides	6 (66)	4.4 ± 2.7	4.1 ± 1.4	7 ± 3	Ns	Ns

*Responders are defined as those responding to the tests at baseline.

T1: time 1 (baseline); T2. time 2 (after 1–2 months INH therapy), T3: time 3 (after 6 months, at therapy completion). IFN: interferon; PHA: Phytohemagglutinin; PPD: purified protein derivative; RD: region of difference; QTF-G: QuantiFERON TB Gold; IFN: interferon; IU: international units. INH: isoniazid; ns: not statistically significant.

#### INH-treated subjects

**Figure 2:**
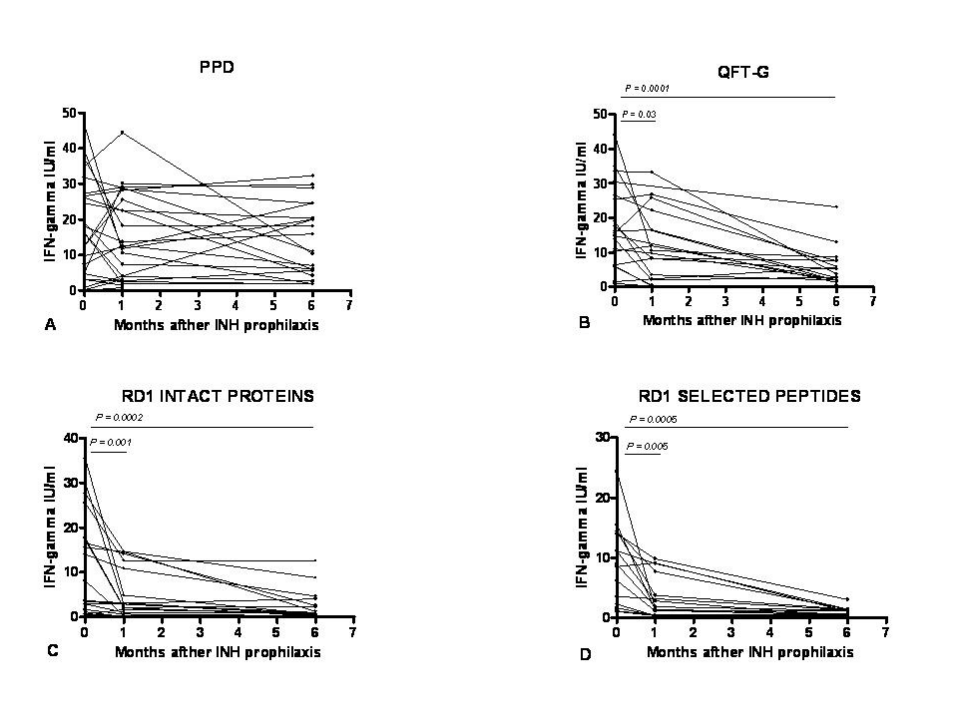
**A-D. INH-treated TST^+^ subjects without a past MTB exposure: time course of *M.tuberculosis*-specific immune response**. Responses to PPD, RD1 intact proteins, RD1 selected peptides and QTF-G in TST^+^ contacts that underwent INH therapy were evaluated over time at time 1 (baseline), time 2 (after 1–2 months of INH therapy), time 3 (therapy completion). Responses to PPD (A) were not significantly affected, unlike those to QTF-G (B), to RD1 intact proteins (C), and to RD1 selected peptides (D), which were found to significantly change over time. PPD: purified protein derivative; RD: region of difference; QTF-G: QuantiFERON TB Gold; IFN: interferon; IU: international units.

The IFN-gamma response to PPD, QTF-G, RD1 intact proteins and selected peptides of the 24 TST^+ ^individuals that started therapy, and that did not report a previous exposure to MTB in the past, is shown in Figure 2 and table 2.

IFN-gamma production in response to PPD was detected in all the individuals studied (24/24, 100%) and did not significantly change over time (Figure 2A and table 2).

In the QTF-G assay, 5 of the 24 contacts did not respond throughout the study period. In the 19 individuals who did show a response, IFN-gamma significantly decreased at time 2 (p = 0.03 vs. time 1) with a 34% reduction over baseline, and a 70% reduction at time 3 (p = 0.0001 vs. time 1) (Figure 2B and table 2). The proportion of positive responses to QTF-G at time 2 (18/19) and at time 3 (18/19) did not differ significantly from that observed at time 1.

Responses to RD1 intact proteins at baseline were negative in 6 of the 24 contacts and remained negative over time. In the 18 responders a significant decrease in IFN-gamma production was noticed: 57% reduction over baseline at time 2, (p = 0.001 vs. time 1) and 79% reduction at time 3 (p = 0.0002 vs. time 1) (Figure 2C and table 2). The percentage of positive responses at time 2 was 77% (14/18) (p > 0.5 vs. time 1) and 61% (11/18) at the end of therapy (p < 0.007 vs. time 1).

Responses to RD1 selected peptides were noticed in 15/24 subjects, while the remaining 9 were negative throughout the study. The 15 responders showed a significant decrease of IFN-gamma production at time 2 (p = 0.005 vs. time 1) with a 63% reduction over baseline, and a 91% reduction at time 3 (p = 0.0005 vs. time 1), as shown in figure 2D and table 2. The percentage of positive responses to RD1 selected peptides was 73% (11/15) at time 2 (p > 0.5 vs. time 1) and 47% (7/15) at the end of treatment (p < 0.002 vs. time 1).

Among these same individuals TST cuticonversion was observed in 9/24. However no differences were observed in the trends of RD1 responses when the results from these individuals were compared with those from the remaining 15 subjects in whom a cuticonversion was not observed (data not shown).

**Figure 3:**
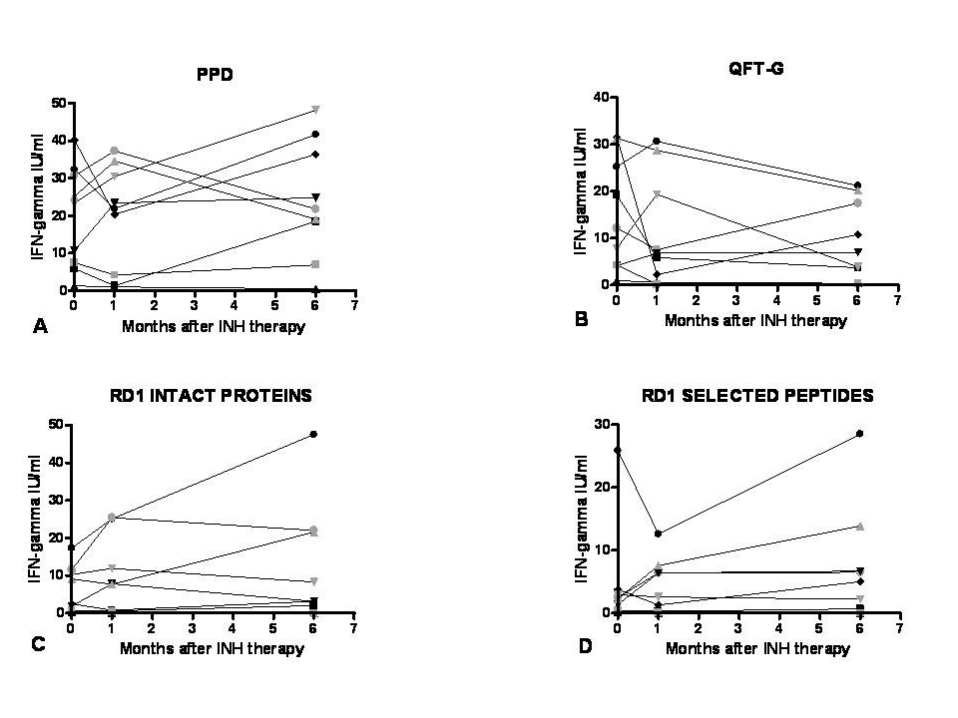
**A-D. INH-treated TST^+^ subjects with a past MTB exposure: time course of *M.tuberculosis*-specific immune response**. Responses to PPD (A), QTF-G (B), RD1 intact proteins (C), and RD1 selected peptides (D) in TST^+^ contacts that underwent INH therapy were evaluated over time at time 1 (baseline), time 2 (after 1–2 months of INH therapy), time 3 (therapy completion) and no statistically significant changes were recorded. PPD: purified protein derivative; RD: region of difference; QTF-G: QuantiFERON TB Gold; IFN: interferon; IU: international units.

It is interesting to note that in the group of the 9 individuals that reported an exposure to MTB in the past and started INH therapy at the time of the present study, the IFN-gamma response to PPD, QTF-G, RD1 intact proteins and selected peptides did not significantly change over time (Figure 3 and table 3). In particular, all 9 individuals responded to PPD, QTF-G, RD1 intact proteins whereas only 6 responded to the selected peptides. Percentages of positive responses remained stable over time for all the stimuli used.

## Discussion

In this pilot study, we show that INH preventive therapy is associated with a significant decrease of the *in vitro *RD1 responses in individuals with LTBI with a recent exposure to MTB, who did not report any exposure in the past. This decrease was observed, although to a different extent, with all RD1 antigen reagents used. It is important to note that the trend of decrease of the test using RD1 selected peptides was particularly marked after 1–2 months of therapy and that the response of the majority became undetectable after six months, while the response to the other RD1 products (overlapping peptides of the QTF-G and RD1 intact proteins) decreased more slowly and fewer negative results were observed after six months of therapy. These differences may be related to the amount and the composition of epitopes covered by the peptides and intact proteins. For example, the peptides employed in the QTF-G cover the whole CFP-10 and ESAT-6 intact proteins (in addition to having a peptide from TB7.7) [12] whereas the peptides used in our assay are few and selected in order to be highly immunogenic [14]. Based on our data, this oligoclonal response appears to be a sensitive tool to monitor MTB replication as well as active TB disease, more so than the polyclonal one against all RD1 epitopes. It is also important to note that among all these recent contacts nine did not respond at the first time of observation to RD1 selected peptides. Five of them did not respond to QTF-G either, and therefore it is likely that these subjects were not infected with MTB, although they were TST^+^. Further investigations are needed to clarify the meaning of these differences. In contrast, no significant variation was observed in individuals with LTBI who, in addition to a recent exposure, had also a past exposure to contagious TB patients. Altogether, these data suggest that RD1-based assays may be a tool to monitor therapy-related changes of MTB-specific immune responses not only in patients with active disease, as previously demonstrated by us and others [15,19-21], but also in individuals with a recently acquired LTBI.

A few studies have been previously published on the effect of LTBI therapy on T-cell responses. Decreasing responses to CFP-10 overlapping peptides, and not to ESAT-6, were found after INH therapy in recently exposed contacts [22] that are comparable to the group of recent contacts evaluated in the present study. However, we did not find differences in the responses to the reagents CFP-10 and ESAT-6 evaluated separately (either proteins or peptides, data not shown). On the other hand, increasing and/or fluctuating IFN-gamma responses were found by Ewer et al. in children exposed for over 9 months to a case of pulmonary TB that underwent therapy (INH and rifampicin) almost a year after exposure [25]. Given the continuous exposure over a considerable period of time described in that paper, it is plausible that LTBI was already established at the time of treatment, with the consequence of a different modulation of T-cell responses compared to our study in which treatment was rapidly started in those without a past contact with MTB. In addition, in this study INH was used as chemotherapy, whereas other studies reported INH plus rifampicin [25] and it is unknown if different chemotherapies may have a different impact on MTB antigen exposure to the immune system [28]. Conversely Pai et al. found unchanging IFN-gamma responses to QTF-G after INH therapy in a population of health care workers (HCW) in India [23]. These subjects, given their continuous exposure to MTB, might have an immune response similar to that of our group of re-infected individuals in which no change of RD1 responses was observed over time, and therefore these results are comparable to ours. These data may indicate that the conflicting results found in the literature may be due, at least in part, to the population selected (recent infection vs. past infection with re-exposure).

The reasons for the different effect of therapy in modulating RD1 responses in recently infected patients vs. those potentially reinfected, observed in the present study, are currently unclear. In several models of infectious diseases it has been shown that the immune response is strictly dependent on pathogen replication and antigenic load [29-31]. In studies on in vitro IFN-gamma response to TB antigens in particular, it has been hypothesized that short incubation assays (as those used in this study) detect responses of partially activated effector T cells that have recently encountered antigens *in vivo*, and can therefore rapidly release IFN-gamma when stimulated *in vitro*. This suggests a correlation between the measure of this response and antigen load [6,11,12]. All the contacts who received INH in this study were exposed to an index TB case who was infected by an INH-sensitive strain, and all these contacts had good adherence to treatment as assessed by interview. Therefore it can be hypothesized that subjects treated early after recent infection may have had a rapidly controlled infection owing to the combination of an effective treatment and an efficient immune response, with a consequent decrease of the RD1-specific effector cells. In contrast, it is likely that contacts with previous untreated LTBI host different populations of mycobacteria, characterised by different growth rates: those originating from the recent infection, which are actively replicating and on which INH is effective, and those in a dormant state that INH is not effective in killing [28]. This pool of dormant bacilli may be the cause of the longer persistence of the T cell response over time and also be responsible for the generation and maintenance of a large population of memory cells, similar to that obtained after several boosts of vaccination; at the time of the present study, reinfection may have caused a rapid and strong effector/memory response caused by the expansion of this pool of central memory cells [32]. However it is possible that this response would decrease at later time points than the 6 months of our study. Therefore, future investigations are needed to evaluate the role of effector and memory T cells on the modulation of this long lasting response not only within, but also beyond the period of treatment. Based on all these observations, although LTBI has been proposed to be a static process [33], our data favour a dynamic model of LTBI, whereby subpopulations of actively replicating bacilli are controlled by the immune response [18,22-25].

Some potential limitations of the present study should be considered. Firstly, we were able to document only 9 cuticonversions among the 24 individuals who did not report a previous exposure to MTB. This limit however is common to previous studies [22,25], and it is important to note that the risk of previous unknown exposures in these individuals is very low because the subjects enrolled were young (median age 21 years) and the majority were born in Italy (68%) where the incidence of TB is low (8 per 100,000 inhabitants in 2004, including 39% of foreign-born individuals) [34]. Secondly, TB exposure was assessed by administering a questionnaire, and thus this information may be affected by recall bias. Thirdly, INH was not administered as directly observed therapy and consequently the evaluation of adherence, performed by patient interview, may have been imprecise. Lastly, generalisation of our results may be limited by the fact that a relatively low number of TST+ subjects were available for follow-up, since many individuals refused further blood drawing.

## Conclusion

In conclusion, our preliminary data suggest the possibility of using RD1 immune responses as surrogate markers of efficacy during LTBI treatment. A larger study is needed to better evaluate the difference in kinetics of the T-cell response after exposure to MTB in those with an already established LTBI vs. those recently infected.

## Abbreviations

• BCG: Bacillus Calmette Guerin

• CFP: culture filtrate protein

• ESAT: early secreted antigenic target

• HIV: Human Immunodeficiency Virus infection

• IFN: interferon

• INH: isoniazid

• IU: international unit

• LTBI: Latent tuberculosis infection

• MTB: *Mycobacterium tuberculosis*

• PHA: Phytohemagglutinin

• PPD: purified protein derivative

• QFT-G: QuantiFERON TB Gold

• RD: region of difference

• RD1: CFP-10 and ESAT-6

• TST: tuberculin skin test

• TB: Tuberculosis

• WBE: whole blood ELISA

## Competing interests

DG, RC, FP and EG have a patent pending on T cell assay based on RD1 selected peptides.

## Authors' contributions

DG designed the study and recruited the adult patients and performed data analysis and wrote the draft of the manuscript, MPP carried out the data base of collected data and helped in the data and statistical analysis, OB and FB and RC carried out the immunological assays, DD and GA helped in the recruitment of adults and children respectively and in the design of the study, CN helped in writing the draft and did the editing and performed data analysis, GI and FP helped in writing the draft of the paper and in the design of the study, EG conceived of the study and participated in its design and performed the statistical analysis and helped to draft the manuscript. The article has not been submitted elsewhere and all co-authors have read and approved the final manuscript with its conclusions.
